# *OGDHL* closely associates with tumor microenvironment and can serve as a prognostic biomarker for papillary thyroid cancer

**DOI:** 10.1002/cam4.3640

**Published:** 2021-01-06

**Authors:** Min Mao, Rong‐zhi Huang, Jie Zheng, Hai‐qi Liang, Wen‐hui Huang, Jing Liu, Jie‐hua Li

**Affiliations:** ^1^ Department of Gastrointestinal Gland Surgery The First Affiliated Hospital of Guangxi Medical University Nanning The Guangxi Zhuang Autonomous Region 530021 China; ^2^ Guangxi Medical University Nanning The Guangxi Zhuang Autonomous Region 530021 China

**Keywords:** *OGDHL*, papillary thyroid cancer, ssGSEA, tumor microenvironment

## Abstract

**Background:**

Papillary thyroid cancer (PTC) is the most common type of thyroid cancer. However, due to the lack of reliable prognostic biomarkers for PTC, overtreatment has been on the rise. Therefore, our research aims to identify new and promising prognostic biomarkers and provide fresh perspectives for clinical decision making.

**Methods:**

The RNA‐seq data and clinical data of PTC samples were obtained from The Cancer Genome Atlas data portal. GSE64912 and GSE83520 datasets were downloaded through the GEOquery R package. The difference in the expression of oxoglutarate dehydrogenase like (*OGDHL*) between PTC and normal tissues was explored by the Wilcoxon test. Kaplan–Meier (KM) and Cox regression analyses were used to further explore the prognostic value of *OGDHL*. The tumor microenvironments of PTC patients were explored based on ssGSEA and Tumor Immune Estimation Resource online database. Gene Set Enrichment Analysis (GSEA) was performed to explore the biological processes associated with *OGDHL*.

**Results:**

The expression level of *OGDHL* in PTC was significantly altered compared to that in normal tissues (*p* < 0.05). Various biological processes associated with *OGDHL* were also explored through GSEA. KM analysis suggested that the low‐*OGDHL* group had a better overall survival [OS, *p* = 3.49e‐03, hazard ratio (HR) = 4.567]. The receiver operating characteristic curve also indicated the favorable prognostic potential of *OGDHL*. Moreover, *OGDHL* was proved to be an independent prognostic indicator in Cox analysis (*p* = 1.33e‐02, HR = 0.152). In the analysis of the tumor microenvironment, the low‐*OGDHL* group showed a lower immune score and stromal score, while tumor purity was higher. The expression of *OGDHL* was also closely correlated with the infiltration of immune cells.

**Conclusion:**

Our study elucidated the influence of *OGDHL* on the prognosis of PTC and demonstrated its potential as a novel biomarker, which would provide new insights into the prognosis monitoring and clinical decision making in PTC patients.

## Graphical abstract

Our study proved that the expression of OGDHL was closely related to the prognosis of PTC, and could be used as a new promising prognostic biomarker for PTC.

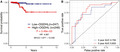

**Abbreviations**
ECM: extracellular matrix
FPKM: fragments per kilobase of exon model per million reads mapped
GEO: Gene Expression Omnibus
GSEA: Gene Set Enrichment Analysis
HCC: hepatocellular carcinoma
MSigDB: Molecular Signatures Database;
OGDHC: oxoglutarate dehydrogenase complex
*OGDHL*: oxoglutarate dehydrogenase like
OS: overall survival
PTC: papillary thyroid cancer
ROS: reactive oxygen species
TC: thyroid cancer
TCGA: The Cancer Genome Atlas

## 1 INTRODUCTION

Papillary thyroid cancer (PTC) is the most common type of thyroid cancer (TC), accounting for approximately 80% of all reported cases of TCs.^1^, ^2^ In recent decades, the development of thyroid cancer diagnostic technology has increased the detection rate of TC rapidly, and the incidence of TC is predicted to replace colon cancer as the fourth leading cancer by 2030.^3^ As the most differentiated TC, the vast majority of PTC patients are at low risk, and do not require immediate and aggressive intervention.^4^ However, due to lack of reliable biomarkers for determining the degree of malignancy of PTC, overtreatment of PTC has been increasing in recent years.^5^ Therefore, it has become essential to find new and reliable prognostic biomarkers for PTC to provide better clinical decisions for patients and reduce overtreatment.

Oxoglutarate dehydrogenase like (*OGDHL*), a subunit of the oxoglutarate dehydrogenase complex (*OGDHC*), is not only the main rate‐limiting component of *OGDHC* for the degradation of glucose and glutamate ^6^, ^7^ but is also involved in the tricarboxylic acid cycle, and in the development and progression of many types of cancers.^8^, ^9^, ^10^, ^11^, ^12^ Sen et al. reported that forced expression of *OGDHL* in cervical cancer can downregulate the protein kinase B (AKT) signaling cascade and reduce the phosphorylation of nuclear factor kappa light‐chain enhancer of activated B cells (NF‐κB) mediated by caspase 3. These activities increase the production of reactive oxygen species (ROS) and lead to cell apoptosis, which, in turn, can inhibit the proliferation and metastasis of cervical cancer cells.^8^ A relevant study also showed that the miR‐214/ TWIST1 (microRNA 214/ Twist‐related protein 1) negative feedback loop mediated by *OGDHL* can inhibit the growth and metastasis of pancreatic cancer.^9^ Shen et al. demonstrated that in hepatocellular carcinoma (HCC), promoter hypermethylation and DNA copy deletion of *OGDHL* were associated with reduced *OGDHL* expression, and silencing of *OGDHL* can further lead to the occurrence and development of HCC by regulating the glutamine metabolism pathway.^10^ Similarly, in breast cancer and sporadic colorectal cancer, abnormal hypermethylation of the *OGDHL* gene promoter also promotes cancer progression.^11^, ^12^ These studies have revealed that *OGDHL* is closely associated with the development and progression of various cancers. However, the relationship between *OGDHL* and PTC still remains poorly understood and needs further investigation.

In the present study, we explore the possible mechanisms closely related to the tumor microenvironment in an attempt to elucidate and comprehensively analyze the relationship between the expression of *OGDHL* and the prognosis of PTC. We hope to provide a reliable base for prognostic prediction and clinical decision‐making optimization in patients with PTC.

## 2 MATERIALS AND METHODS

### 2.1 Differential expression of *OGDHL* between PTC and normal tissues

The fragments per kilobase of exon model per million mapped reads (FPKM) RNA‐seq data and clinical data of 493 PTC samples and 58 non‐tumor samples were obtained from The Cancer Genome Atlas (TCGA) data portal (https://cancergenome.nih.gov/). The Ensembl IDs were converted into gene symbols based on the Genome Reference Consortium human (GRCh38.98) genome file acquired from the Ensembl database (http://asia.ensembl.org/index.html). The expression file for *OGDHL* was then extracted for further analysis. The Wilcoxon test was performed to ascertain the differential expression between PTC and normal tissues. Additionally, the expression profiles of GSE64912 (with 18 primary PTC samples and 4 non‐tumor samples) and GSE83520 (with 12 primary PTC samples and 12 non‐tumor samples) were also downloaded from Gene Expression Omnibus (GEO. http://www.ncbi.nlm.nih.gov/geo/) and the results were verified using the Wilcoxon test or paired Wilcoxon test through the GEOquery R package.

### 2.2 Survival outcomes and multivariate COX analysis

The prognostic value of *OGDHL* was estimated through survival analysis using a log2 (FPKM value +1) data format. We used Kaplan–Meier (KM) analysis to confirm statistical significance, and the cutoff value was determined from its median value. Univariate and multivariate Cox analyses were performed to compare the survival impact of *OGDHL* with other clinical characteristics. The receiver operating characteristic (ROC) curves within 3‐years and 5‐years were established to further analyze the prognostic potential of *OGDHL* in PTC using the timeROC R package.

### 2.3 *OGDHL* mRNA level and tumor microenvironment

The cell infiltration score of the tumor microenvironment between high and low‐*OGDHL* groups was further analyzed in order to explore the biological mechanism of *OGDHL* in PTC. The tumor microenvironment is generally composed of a variety of cell types, including immune cells, mesenchymal cells, endothelial cells, inflammatory mediators, and extracellular matrix (ECM) molecules.^13^ In this study, the ESTIMATE algorithm was used to determine the quantitative cell score of the tumor microenvironment for each PTC patient. Furthermore, the difference between the high and low‐*OGDHL* groups was further compared using the Wilcoxon test.

Additionally, the tumor immune cell infiltrating abundance of each PTC patient was calculated using the Tumor Immune Estimation Resource online database (https://cistrome. shinyapps.io /timer) to further explore the immune‐related mechanisms between *OGDHL* expression and the prognosis of PTC patients.^14^ Pearson correlation analysis was used to further evaluate the relationship between the infiltrating abundance of each component and *OGDHL* expression. A *p* value < 0.05 and /R/ >0.15 indicated that a correlation existed between *OGDHL* expression and immune cell infiltration.

### 2.4 Gene Set Enrichment Analysis (GSEA)

GSEA was used to explore the signaling pathways and biological processes which were differentially activated between the high and low‐*OGDHL* groups. We also identified an ordered list of genes using the edgeR R package and executed GSEA on the gene with adjusted *p* < 0.05. The number of random sample permutations was set at 1000, while the minimum size of the gene set was stipulated at 350.

### 2.5 Statistical analysis

All statistical analyses were performed using the R software (version 3.6.1) (http://www.r‐project.org/) and the corresponding R packages. The KM analysis and Cox regression analyses were completed using the survival R package. The Area Under Curve (AUC) of the ROC curve was calculated using the timeROC package for R. The c5.bp.v6.2.entrez.gmt file from the Molecular Signatures Database (MSigDB, http://software.broadinstitute.org/gsea/index.jsp) was downloaded for the GSEA and was completed by using the clusterProfiler R package.

## 3 RESULTS

### 3.1 Differential expression of *OGDHL* between PTC and normal samples

**FIGURE 1:**
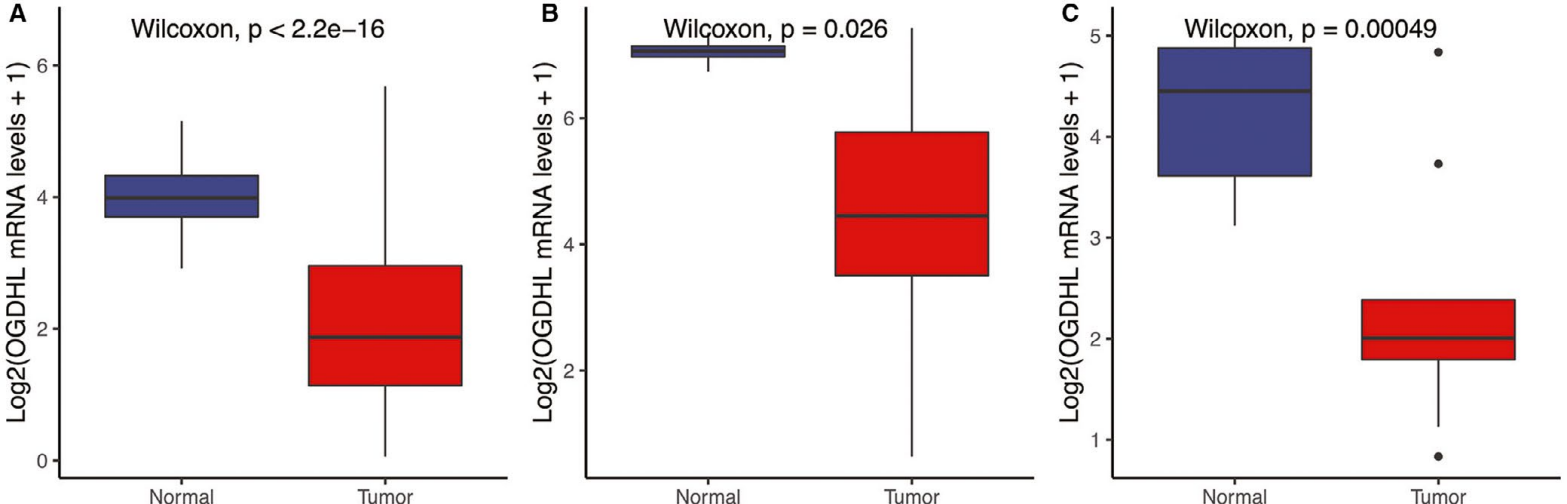
Boxplots of the analysis of *OGDHL* expression levels between PTC and normal tissues. The results indicate that expression of *OGDHL* is lower in PTC compared to that in normal tissues (*p* < 0.05). (A) TCGA RNA‐seq. (B) GSE64912. (C) GSE83520.

*OGDHL* expression in the TCGA RNA‐seq, GSE83520, and GSE64912 datasets showed significant statistical differences (Figure 1). *OGDHL* was lower in PTC samples than in the normal samples (*p* < 0.05, Figure 1A‐1C).

### 3.2 Survival outcomes and multivariate Cox analysis

**FIGURE 2:**
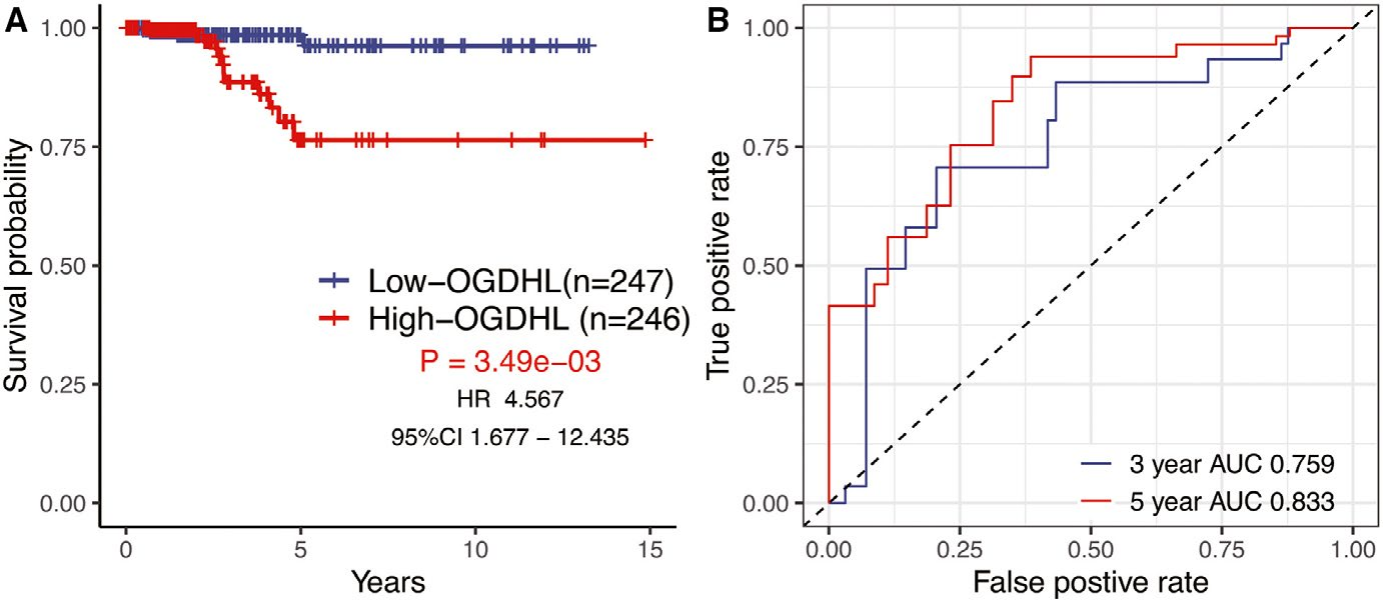
(A) Impact of *OGDHL* expression on overall survival in PTC patients in TCGA RNA‐Seq cohort. (B) Time‐dependent receiver operating characteristic (ROC) curve analysis of *OGDHL*.

We observed that patients with high‐*OGDHL* expression were correlated with worse overall survival in the KM analysis [*p* =3.49e‐03, hazard ratio (HR) = 4.567, 95% confidence interval (CI) = 1.677–12.435] compared to patients with low‐*OGDHL* expression (Figure 2A). In the meantime, the time‐dependent ROC curve analysis indicated that *OGDHL* had a promising prognostic potential for predicting overall survival in PTC (area under the curve, AUC_3‐year_ = 0.759, AUC_5‐year_ = 0.833) (Figure 2B). In addition, comparison with other clinical features by univariate and multivariate Cox regression analyses also revealed that *OGDHL* was independently associated with overall survival (OS) (HR = 6.576, *p* = 1.33e‐02) (Table 1).

**TABLE 1:** The results of univariate and multivariable Cox regression analyses.

Characteristics	Univariate Cox analysis		Multivariate Cox analysis	
	HR (95%CI)	*p* Value	HR (95%CI)	*p* Value
Age	1.144 (1.090–1.202)	6.87e−08	1.153 (1.088–1.222)	1.64e−06
Gender	2.080 (0.753–5.743)	1.58e−01	1.173 (0.310–4.447)	8.14e−01
Tumor status	2.248 (1.247–4.050)	7.04e−03	1.088 (0.393–3.018)	8.71e−01
Lymph node metastasis	1.562 (0.769–3.170)	2.17e−01	1.655 (0.651–4.209)	2.90e−01
Distant metastasis	0.912 (0.545–1.525)	7.25e−01	0.706 (0.35–1.4240)	3.31e−01
Pathological stage	2.596 (1.629–4.137)	5.97e−05	1.080 (0.407–2.870)	8.77e−01
Residual tumor	1.763 (1.162–2.675)	7.65e−03	1.501 (0.855–2.634)	1.58e−01
*OGDHL* (Low/High)	4.712 (1.505–14.752)	7.77e−03	6.576 (1.482–29.184)	1.33e−02

Abbreviation: HR, hazard ratio; CI, confidential interval; OGDHL, Oxoglutarate dehydrogenase like

### 3.3 High‐ and low‐*OGDHL* groups displayed different tumor microenvironments

**FIGURE 3:**
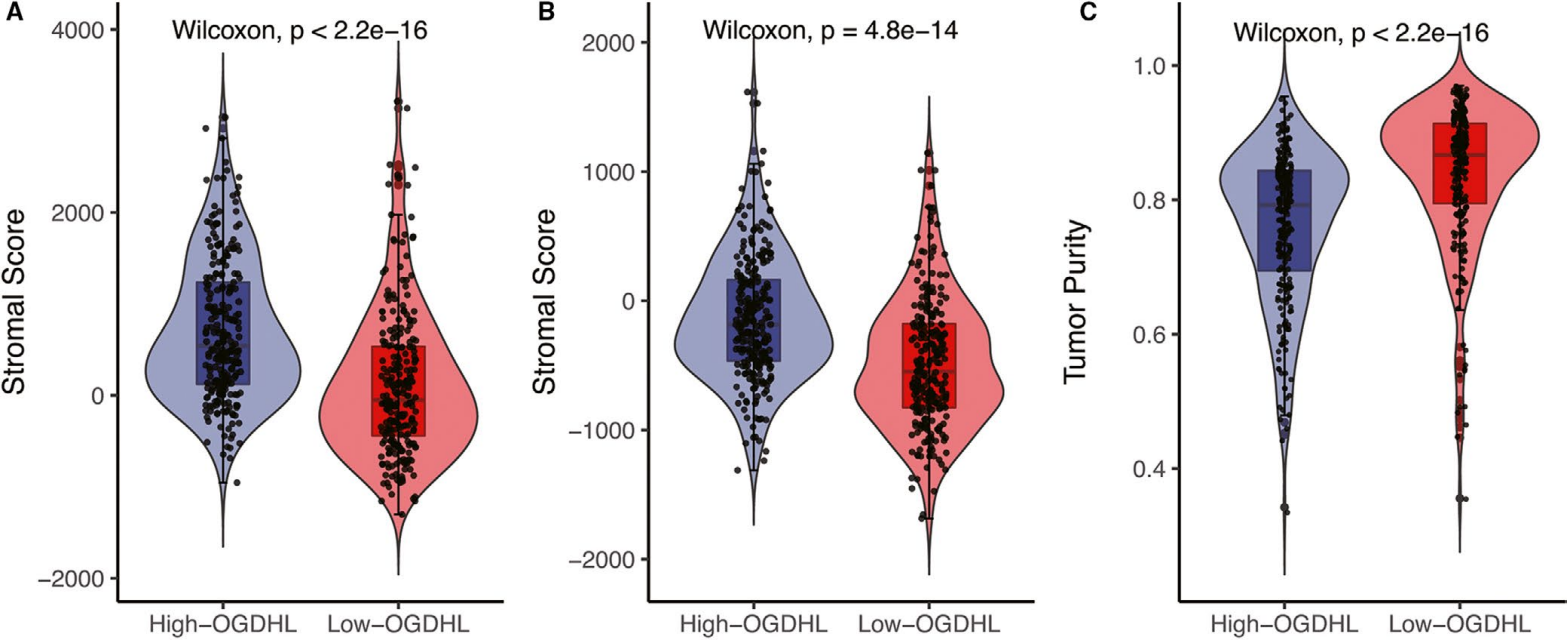
The relationships between the expression of *OGDHL* and immune score, stromal score, and tumor purity of tumor microenvironment. The low‐*OGDHL* group demonstrated (A) lower immune score, (B) lower stromal score, and (C) higher tumor purity than the high‐*OGDHL* group.

The results from the ESTIMATE algorithm showed that the immune score, stromal score, and tumor purity of the tumor microenvironment were statistically different between the high and low‐*OGDHL* groups. The low‐*OGDHL* group had a lower immune score, lower stromal score, but a higher tumor purity than the high‐*OGDHL* group (Figure 3A‐3C, *p* < 0.05). These observations indicated that a difference in *OGDHL* expression in PTC patients results in a difference in the respective tumor microenvironments.

**FIGURE 4:**
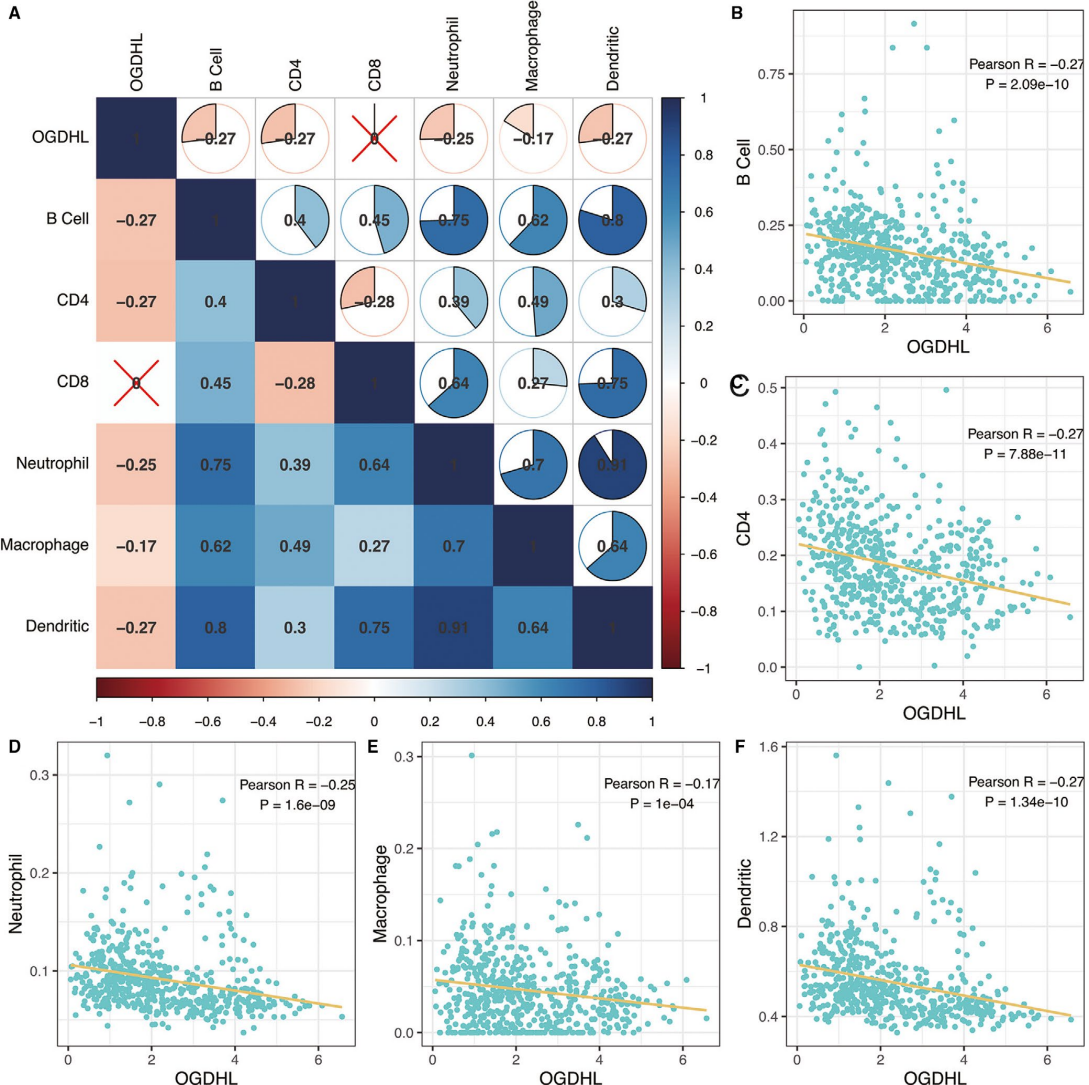
(A) The correlation between the eight immune cell components and *OGDHL*. The B cell, CD4+, Neutrophil, Macrophage, and Dendritic were negatively correlated with *OGDHL* according to our criterion. (B) Relationship between B cells and *OGDHL*. (C) Relationship between CD4+ and *OGDHL*. (D) Relationship between neutrophils and *OGDHL*. (E) Relationship between macrophages and *OGDHL*. (F) Relationship between dendritic cells and *OGDHL*.

In addition, we further analyzed the relationship between the immune cell components of the tumor immune microenvironment and the expression of *OGDHL*. Pearson correlation analysis showed that immune cell infiltration in the tumor microenvironment was associated with expression of *OGDHL* (Figure 4A, *p* < 0.05). The B cells, CD4+ cells, neutrophils, macrophages, and dendritic cells were all negatively correlated with *OGDHL* according to our criterion (Figure 4B‐F, *p* < 0.05).

### 3.4 The different immune‐related biological processes associated with *OGDHL*

**FIGURE 5:**
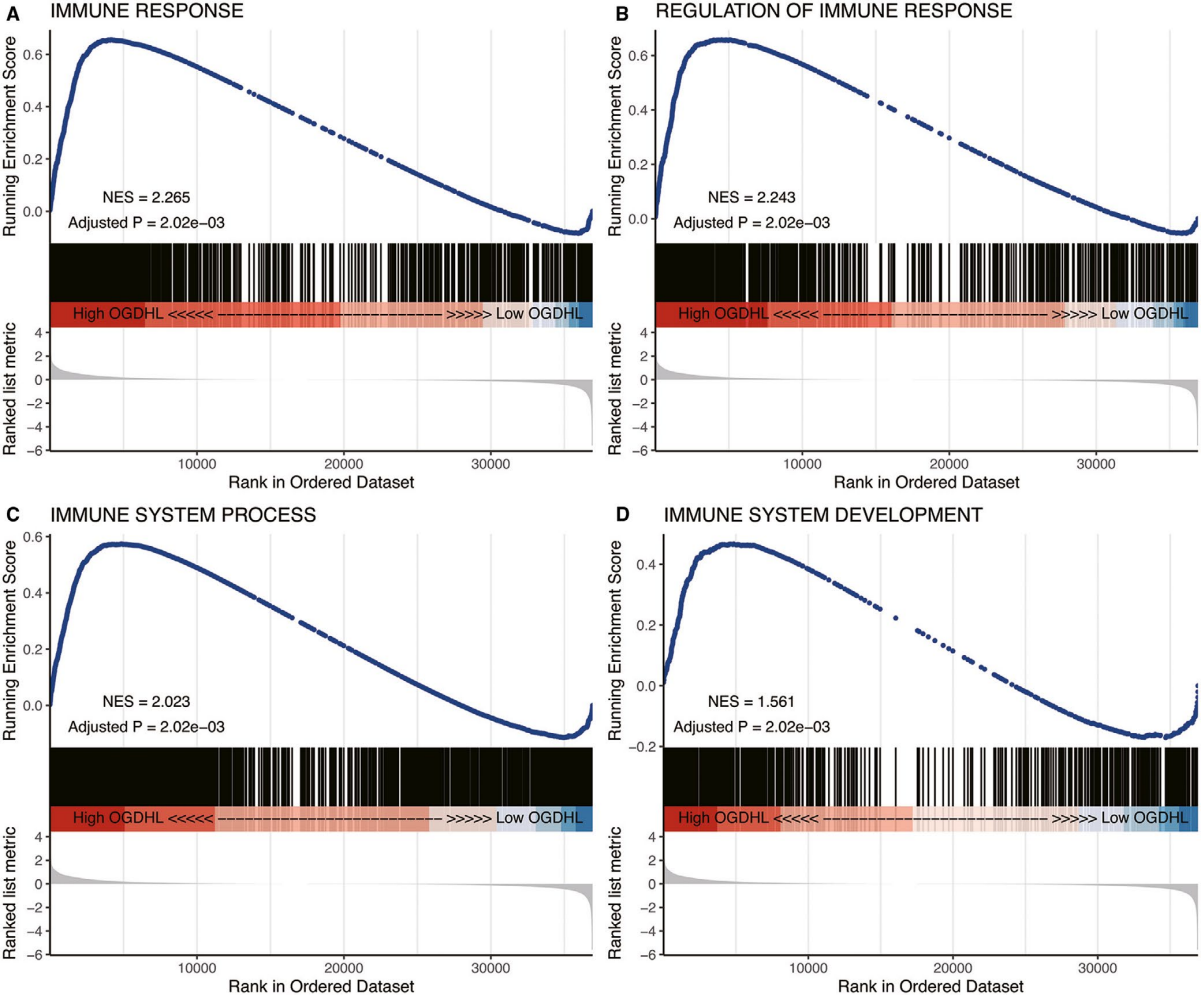
Parts of Gene Set Enrichment Analysis (GSEA) results indicating that various immune‐associated biological processes are also associated with *OGDHL*, including (A) immune response, (B) regulation of immune system, (C) immune system processes, and (D) immune system development.

GSEA revealed that various immune‐associated biological processes were enriched, including immune response, immune system process, regulation of immune system response, and immune system development (Figure 5A‐5D, S1).

## 4 DISCUSSION

OGDHL, as the main rate‐limiting component of 2‐oxoglutarate dehydrogenase complex (OGDHC) for glucose and glutamic acid degradation, plays an important role in the development of a wide variety of cancers ^7^ and tumors by influencing cell cycle arrest,^9^ cell apoptosis,^8^, ^15^ and energy metabolism.^10^ Studies have shown that methylation of the *OGDHL* promoter contributes to the development of many cancers such as breast cancer ^11^ and colon cancer.^16^ Our analysis also revealed significant changes in *OGDHL* expression in PTC samples, thereby justifying our pursuit in elucidating the role of *OGDHL* in PTC.

It is worth noting that as per the KM analysis, the low‐*OGDHL* group had a better OS than the high‐*OGDHL* group, while the differential expression analysis showed a decreased level of *OGDHL* expression in PTC samples than that in the normal tissues. This observation was rather remarkable as it suggested that *OGDHL* may not only play different roles in the occurrence and progression of PTC but may also be closely related to the unique molecular mechanisms and functions of *OGDHL* affecting PTC. In addition, *OGDHL* was also determined as an independent prognostic marker of PTC by Cox analysis and ROC curve analysis. Therefore, it is evident that *OGDHL* has a novel and promising potential to be employed as a reliable prognostic biomarker for PTC patients.

We performed GSEA to explore the biological mechanism of *OGDHL* acting on PTC and demonstrated that diverse biological processes endowed with different *OGDHL* expression levels were closely related to tumor immunity, including immune response, regulation of the immune system, immune system processes and overall immune system development. The immune system is known to coordinate with other systems for maintaining the internal stability and physiological balance of the body,^17^, ^18^ but in‐depth studies on tumor antigens have revealed that the immune response—a physiological process employed by the immune system to eliminate antigens—plays an increasingly important role in the development of tumors.^19^, ^20^, ^21^ The regulation of the immune system also acts on many aspects of the immune response process such as maintaining the immune responses at an appropriate level and avoiding immune response disorder, which, in turn, are essential for sustaining the stability of the body's internal environment and influencing the development of cancers.^18^, ^22^ Existing studies have demonstrated the increasing importance of immunity in PTC, and various immunotherapies have also been applied in PTC.^23^, ^24^, ^25^ These observations are consistent with our analysis that the expression of *OGDHL* in PTC is closely related to biological immune processes. Therefore, we believe that our preliminary analysis can broaden our understanding of the influence of *OGDHL* on PTC immunology and provide a perspective for further exploration.

Tumor development is influenced not only by its characteristics but also by the unique tumor microenvironment. Increasing evidence indicates that the tumor microenvironment plays an important role in predicting tumor development and prognosis.^26^, ^27^, ^28^ Our findings from the ESTIMATE algorithm also indicates that groups with different *OGDHL* expression levels show different tumor microenvironments, thereby further supporting the potential of *OGDHL* as a PTC prognostic marker closely related to the PTC tumor microenvironment.

Another important aspect of the tumor microenvironment is immune cell infiltration, which deeply impacts the outcomes of PTC.^29^ Some existing studies have shown that neutrophils are involved in promoting tumor proliferation, metastasis, and tumor angiogenesis,^30^, ^31^ while others suggest that neutrophils plays an anti‐tumor role through the blocking of TGF‐β or the activation of cytokines.^32^, ^33^ In our study, Pearson correlation analysis also showed that neutrophil infiltration can be beneficial to the prognosis of PTC. Ugolini et al. proved that the infiltration of dendritic cells was associated with a decrease in the invasion rate and the prolongation of disease‐free survival of PTC,^34^ which is consistent with our analysis. In addition, B cells have been observed to accumulate in primary thyroid tumors, producing antibodies and presenting antigens to T cells, thereby enhancing the immune response, and although there was no evidence collocating B‐cell infiltration with disease severity, the positive influence of B cells in eliminating cancer was confirmed.^35^, ^36^, ^37^ M1 macrophages, similar to B cells, reportedly promote the cytotoxic natural killing ability and T‐cell responses by presenting antigens, which play a role in cancer inhibition.^38^ Bastman et al. found that CD8+T cells infiltrated at different statuses of thyroid cancer, such as locally invasive differentiated, anaplastic, and distant metastases of thyroid cancer, which can indirectly protect the patients.^29^ In addition, CD4+T cells often differentiate into helper T cells, producing cytokines, and cooperating with CD8+T cells to kill cancer cells yy.^37^ Therefore, the interactions between *OGDHL* and tumor microenvironment immune cell infiltration can be a potential mechanism for correlating *OGDHL* expression with prognosis in PTC. These observations can be further explored for a holistic understanding of the nuances of PTC microenvironment immune cell infiltration.

## 5 CONCLUSION

This study not only comprehensively discusses the effect of *OGDHL* on the prognosis of PTC but is also, to our knowledge, the first to elaborate on the relationship between *OGDHL* and PTC. However, there are certain limitations that need to be considered in our study. First, due to lack of in vitro experiments, the specific mechanism of *OGDHL* in PTC still needs to be clinically verified. Second, the transcriptome data used in this study were all from core samples of tumor tissue, so the influence of the microenvironment in different tumor regions on tumor progression could not be considered. Therefore, a well‐designed clinical trial is required to further validate our observations, although the mode of action of *OGDHL* in PTC has been effectively elucidated through various methods, which would provide new insights for prognosis monitoring and clinical decision making in PTC patients.

## 6 ETHICAL APPROVAL STATEMENT

Not applicable.

## CONFLICT OF INTERESTS

All authors have read and approved to submit it to your journal. There are no conflicts of interest of any authors in relation to the submission.

## AUTHOR CONTRIBUTIONS

All authors read and approved the final manuscript.

## Data Availability

R 3.6.1(http://www.r‐project.org/) was an open source software. The RNA‐FPKM data and clinical data of PTC samples came from the TCGA data portal (https://cancergenome.nih.gov/). GSE64912 and GSE83520 gene expression profiles were downloaded through GEOquery R package. The GRCh38.98 genome file could acquire from the Ensembl database (http://asia.ensembl.org/index.html).
